# Small airway dysfunction in smokers with stable ischemic heart disease

**DOI:** 10.1371/journal.pone.0182858

**Published:** 2017-08-28

**Authors:** Claudia Llontop, Cristina Garcia-Quero, Almudena Castro, Regina Dalmau, Raquel Casitas, Raúl Galera, Alberto Iglesias, Elisabet Martinez-Ceron, Joan B. Soriano, Francisco García-Río

**Affiliations:** 1 Service de Pneumologie et Réanimation Médicale, Hôpital Pitié-Salpêtrière, Paris, France; 2 Servicio de Neumología, Hospital Universitario La Paz, IdiPAZ, Madrid, Spain; 3 Servicio de Cardiología, Hospital Universitario La Paz, IdiPAZ, Madrid, Spain; 4 CIBER de Enfermedades Respiratorias (CIBERES), Madrid, Spain; 5 Instituto de Investigación Hospital Universitario de la Princesa (IISP), Madrid, Spain; 6 Facultad de Medicina, Universidad Autónoma de Madrid, Madrid, Spain; National and Kapodistrian University of Athens, SWITZERLAND

## Abstract

**Background:**

A higher prevalence of airflow limitation (AL) has been described in patients with ischemic heart disease (IHD). Although small airway dysfunction (SAD) is an early feature of AL, there is little information about its occurrence in IHD patients. Our objective was to describe the prevalence of SAD in IHD patients, while comparing patient-related outcomes and future health risk among IHD patients with AL, SAD and normal lung function.

**Methods:**

In 118 consecutive smoking patients with stable IHD, comorbidities, utilization of healthcare resources, current treatment, blood biochemistry and health status were recorded. SAD was evaluated by impulse oscillometry, and pre- and post-bronchodilator spirometry was performed.

**Results:**

The prevalence of AL and SAD were 20.3 (95% CI, 13.1–27.6%) and 26.3% (95% CI, 18.3–34.2%), respectively. Compared to the normal lung function group, patients with SAD and without AL had lower spirometric values, poorer quality of life and higher levels of C-reactive protein (CRP), as well as increased cardiovascular risk and more vascular age. In patients with normal spirometry, the presence of SAD was independently associated with pack-years, HDL-cholesterol and CRP levels.

**Conclusion:**

In patients with IHD, the presence of SAD is common and that it is associated with reduced health status and increased future cardiac risk.

## Introduction

Cigarette smoke is a major contributor to the pathogenesis of both chronic obstructive pulmonary disease (COPD) and ischemic heart disease (IHD) [1,2], two of the most common causes of death in the world [3]. Some studies have reported a higher prevalence of airflow limitation compatible with COPD in patients with cardiovascular disease than in the general population [4–6], particularly in patients with IHD [4]. Thus, it has been suggested that the risk of IHD is further increased in smokers who have developed COPD, and that COPD might be an independent risk factor for IHD [7]. Recently, the ALICE study has demonstrated that airflow limitation affects almost one-third of former or current smoking patients with IHD, and that it is associated with increased symptom burden, reduced health status, and increased health risk [8].

Because the small airways are the major site of obstruction in patients with COPD,[9] the term small airway dysfunction (SAD) has been used in reference to abnormalities occurring secondary to cigarette smoking as an early feature of COPD that might also account for the initial progression of airflow limitation in these patients [9–11]. To date, there is no specific information about the prevalence of SAD in smoking patients with IHD who they have not yet developed COPD, nor of its clinical or prognostic implications. Among other reasons, the difficulty to specifically study this portion of the airways probably justifies this lack of information.

However, in recent years, the impulse oscillometry system (IOS) has been increasingly used to evaluate small airway function. This simple, noninvasive and sensitive technique utilizes pressure applied to the airways at a range of frequencies, and components of respiratory impedance are measured, including resistance and reactance [12]. Resistance at 5 Hz (R5) and 20 Hz (R20), respectively, represent total airway resistance and proximal airway resistance. The difference between these two values can be calculated (R5-R20), with higher values suggesting the presence of SAD [12].

We hypothesized that SAD is frequent in smokers with IHD and that it may also have clinical and prognostic implications for patients, though perhaps not as pronounced as COPD. The objectives of this study were to describe the prevalence of SAD in smoking patients with IHD and compare patient-related outcomes and future health risk among patients with airflow limitation (AL), SAD without AL and normal lung function.

## Material and methods

### Study subjects

118 patients with IHD were consecutively recruited from an academic medical center (Hospital Universitario La Paz-Cantoblanco, Madrid, Spain). Inclusion criteria were: aged 40 years and older; history of smoking >10 pack-years; and current diagnosis of stable IHD (including history of acute myocardial infarction (MI) or angina pectoris) in accordance with European Society of Cardiology guidelines [2]. Exclusion criteria were recent surgery or MI (within 1 month), lower respiratory tract infection or pneumothorax (within 2 months), or stroke (12 months); any other contraindication for spirometry [13]; or a pre-existing condition which, in the opinion of the researcher, would compromise the safety of the subject in this study. Medication was kept constant during the course of the study. The study was approved by the institutional ethics committee (Hospital Universitario La Paz, Madrid, Spain; PI-1261) and each participant gave written informed consent.

### Clinical evaluation

Anthropometric and demographic characteristics of patients were recorded, as well as any cardiovascular and respiratory comorbidities, utilization of healthcare resources in the last year, and current treatment. The severity of angina was graded according to the Canadian Cardiovascular Society (CCS) grading of angina pectoris, and the New York Heart Association (NYHA) functional classification was determined. Respiratory symptoms were assessed using a standardized American Thoracic Society questionnaire [14] and the COPD Population Screener [15]. Dyspnea was quantified by the Medical Research Council scale (MRC), and body mass index, obstruction, dyspnea (BOD) and ADO [16] indices were determined.

### Lung function tests

All assessments were performed by a single investigator (C.Ll.), using a MasterScreen (Viasys Healthcare, Würzburg, Germany) with a Lilly-type heated screen pneumotachograph. In order to avoid the effects of forced expirations on respiratory smooth muscle tone, impulse oscillometry (IOS) was performed prior to spirometry. A volume calibration at different flow rates was performed daily using a 3-L syringe, and the accuracy of resistance measurements was confirmed daily using a standard 0.20 kPa/l/s resistance mesh.

IOS was performed according to standard guidelines [17]. Participants were asked to wear a nose-clip and were seated with their head in a neutral position during tidal breathing with their lips sealed tightly around the mouthpiece, and while firmly supporting their cheeks with their hands. Studies were performed before inhalation of salbutamol and three acceptable trials (coherence values at 5 and 20 Hz ≥ 0.7 and 0.9, respectively), each lasting 30 s, were performed. The average of three acceptable and reproducible tests (variability < 10%) was used for the analysis. Respiratory resistance at 5 and 20 Hz (R5 and R20, in kPa/l/s) were used as indices of total and proximal airway resistance, respectively, and the fall in resistance from 5 to 20 Hz (R5-R20, in kPa/l/s) was considered an index for the resistance of peripheral airways. Moreover, reactance at 5 Hz (X5, in kPa/l/s) was considered a representative marker of peripheral airway abnormalities. Reactance area (AX) was calculated as the area under the reactance curve from 5 Hz to the resonant frequency, since it is complementary to frequency dependence of resistance and also reflects peripheral airway function. European IOS predicted values of Oostveen [18] were used to calculate the upper limits of normal for R5 and R5–20.

Pre- and post-bronchodilator spirometry was performed according to the standards of the American Thoracic Society/European Respiratory Society [19], and Global Lung Function Initiative reference values were used [20].

### Patients’ classification

According the results of lung function tests, patients were classified in three mutually exclusive groups: airflow limitation, SAD without airflow limitation, and control. Airflow limitation (AL), compatible with a diagnosis of COPD, was defined as a post-bronchodilator FEV_1_/FVC <lower limit of normal, and staged according to the Global Initiative for Obstructive Lung Disease (GOLD) report [21]. SAD was defined as R5 and R5-R20 above upper limit of normal [22]. In the absence of airflow limitation and SAD, patients were considered as control.

### Quality of life and future health risk

Health status was measured by the COPD Assessment Test (CAT) and the SF-12, a generic quality of life instrument. Standard blood chemistry was performed, including low-density lipoprotein cholesterol, high-density lipoprotein (HDL) cholesterol, total cholesterol, triglycerides, glycated hemoglobin (HbA1c), high-sensitivity C-reactive protein (hs-CRP), pro-brain natriuretic peptide (pro-BNP), and fibrinogen. To assess future health risk, the cardiovascular disease risk score and the heart/vascular age were calculated [23].

### Statistical analysis

In order to estimate the sample size, a population percentage of SAD around 25% was assumed. A sample size of 118 subjects was considered sufficient to estimate this prevalence with a 95% confidence, ± 7.8 percent units of precision and with no replacement rate.

The normal distribution of variables was tested using the Kolmogorov-Smirnov test. Continuous variables were expressed as mean ± standard deviation or median (interquartile range), depending on their normality distribution. Categorical variables were reported as absolute numbers and percentages. Comparisons between groups (AL, SAD without AL and control) were performed using analysis of variance with Bonferroni post hoc test for continuous variables and the Chi-squared test for categorical variables.

The relationships between variables were determined using Spearman’s correlation. To examine risk factors for SAD in subjects without airflow limitation, odds ratios were calculated by logistic regression. We developed multiple logistic regression models with adjustment for sex, age, body mass index, CCS angina grade and NYHA functional class.

Statistical significance was assumed for *p*<0.05. All analyses were performed using the Statistical Package for the Social Sciences, version 14.0 software (SPSS Inc., Chicago, IL).

## Results

**Fig 1:**
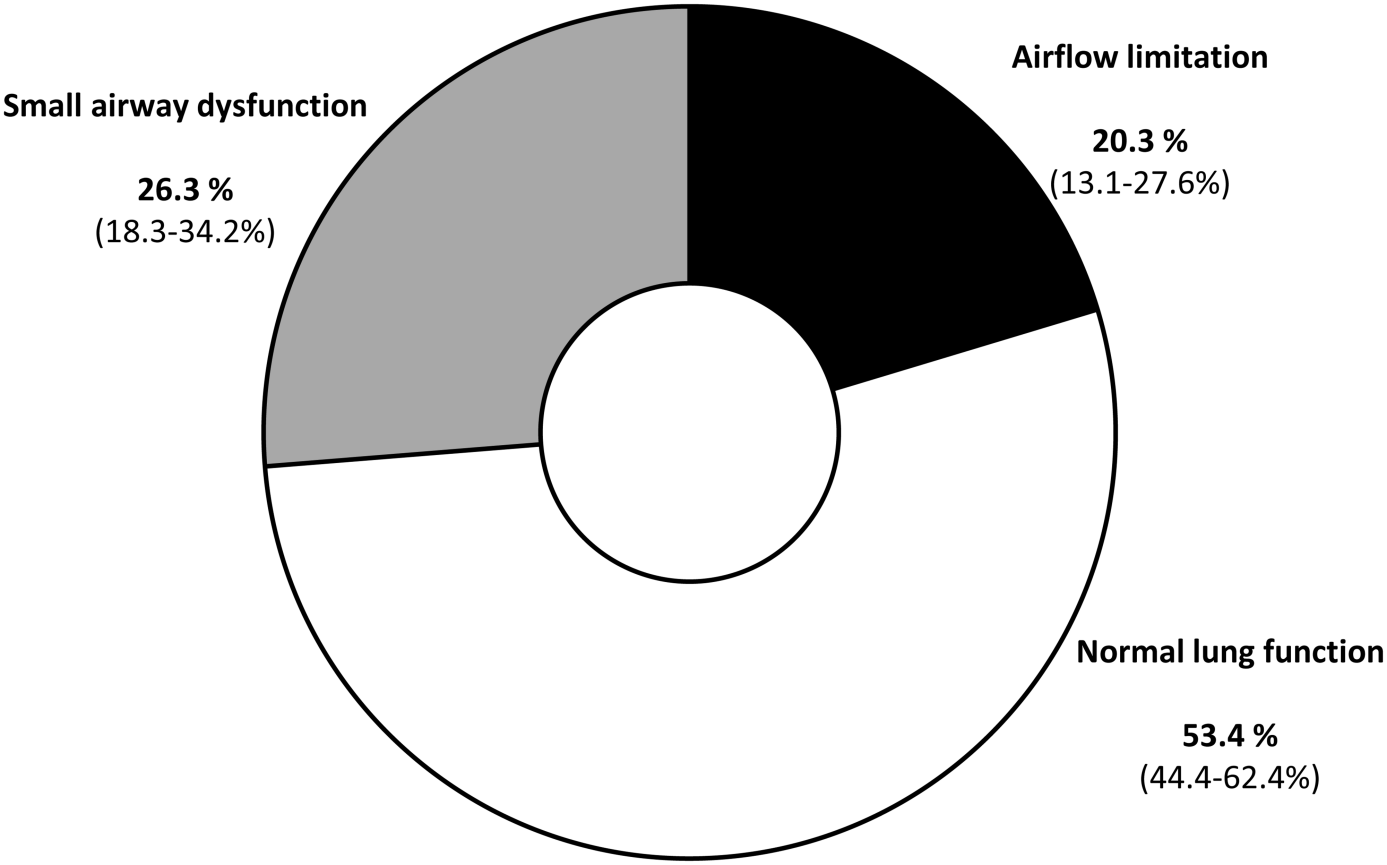
Prevalence of airflow limitation and small airway dysfunction in patients with ischemic heart disease. 95% confidence intervals are shown in parentheses.

Airflow limitation was present in 24 of the 118 patients (prevalence: 20.3% [95% confidence interval, 13.1 to 27.6%]), while SAD without AL was detected in 31 subjects (prevalence: 26.3% [95% CI, 18.3 to 34.2%]) (Fig 1).

Table 1 shows the comparison of the general characteristics between patients with AL, SAD without AL and normal lung function. The three groups were homogeneous for the anthropometric characteristics and no differences were detected in comorbidities, angina grading scale, functional classification or current medication (all p>0.05). The only distinction was that the patients with AL showed a higher intensity of exposure to tobacco than the control subjects (p<0.05), and the level of dyspnea was also higher in patients with AL than in the other two groups.

**Table 1:** General characteristics of the study subjects*.

	Control group	Small airway dysfunction group	Airflow limitation group	*p*-value
N	63	31	24	-
Males, n (%)	63 (100%)	29 (94%)	23 (96%)	0.149
Age, years	61 ± 8	58 ± 8	59 ± 7	0.121
Body mass index, kg/m^2^	27.4 ± 4.0	28.3 ± 4.2	27.5 ± 4.1	0.104
Hip-waist ratio	0.96 ± 0.04	0.98 ± 0.07	0.96 ± 0.04	0.221
Smoking habit, n (%)				<0.001
Current smoker	12 (19%)	8 (26%)	16 (70%)	
Former smoker	51 (81%)	23 (74%)	7 (30%)	
Pack-years	41.7 ± 19.2	51.0 ± 26.2	63.4 ± 27.2 #	0.001
Comorbidities				
Left congestive heart failure	32 (51%)	17 (55%)	19 (79%)	0.053
Arrhythmia	3 (5%)	1 (3%)	3 (13%)	0.299
Hypertension	32 (51%)	13 (42%)	14 (58%)	0.475
Diabetes	18 (29%)	7 (23%)	5 (21%)	0.695
Metabolic syndrome	49 (78%)	28 (90%)	20 (83%)	0.323
CCS Angina Grading Scale				0.613
Class 0	55 (87%)	28 (90%)	19 (79%)	
Class I	7 (11%)	3 (10%)	5 (21%)	
Class II	1 (2%)	0	0	
NYHA Functional Classification				0.231
Class I	31 (49%)	13 (42%)	6 (25%)	
Class II	23 (37%)	10 (32%)	11 (46%)	
Class III	9 (14%)	8 (26%)	7 (29%)	
Left ventricle ejection fraction, %	53.1 ± 11.5	55.2 ± 10.1	54.6 ± 10.8	0.733
Systolic blood pressure, mmHg	123 ± 17	129 ± 17	122 ± 18	0.279
Diastolic blood pressure, mmHg	74 ± 9	76 ± 11	73 ± 11	0.477
Heart rate, bpm	62 ± 10	65 ± 10	64 ± 9	0.360
History of lung disease				
COPD	1 (2%)	1 (3%)	2 (8%)	0.298
Asthma	0	1 (3%)	2 (8%)	0.084
GOLD screener	3.41 ± 1.10	3.26 ± 0.86	3.42 ± 0.83	0.755
Respiratory symptoms				
Dyspnea MRC grade	0.48 ± 0.51	0.59 ± 0.60	0.97 ± 0.53 ‡§	0.001
Chronic cough, n (%)	10 (16%)	5 (16%)	7 (29%)	0.346
Chronic sputum, n (%)	10 (16%)	9 (10%)	4 (17%)	0.680
Wheezing, n (%)	17 (27%)	10 (32%)	13 (54%)	0.056
Airflow limitation severity				
Mild (GOLD I)	0	0	10 (43%)	-
Moderate (GOLD II)	0	0	13 (57%)	
Cardiac medications				
Antiplatelet drugs	62 (98%)	31 (100%)	24 (100%)	0.644
Anti-thrombotic drugs	55 (87%)	28 (90%)	24 (100%)	0.182
Lipid modifying agents	60 (95%)	31 (100%)	22 (92%)	0.300
ACE inhibitors	24 (38%)	12 (39%)	7 (29%)	0.708
Beta-blockers	56 (89%)	29 (94%)	22 (92%)	0.753
Angiotensin II receptor blockers	9 (14%)	5 (16%)	2 (8%)	0.683
Diuretics	13 (21%)	14 (45%)	11 (46%)	0.016
Calcium channel blockers	4 (6%)	1 (3%)	2 (8%)	0.714
Respiratory medications				
Short-acting ß-agonists	0	0	1 (4%)	0.139
Long-acting muscarinic antagonist	0	2 (7%)	2 (8%)	0.087

*Definition of abbreviations*: ACE = angiotensin-converting enzyme; BNP = brain-type natriuretic peptide; CCS = Canadian Cardiovascular Society; COPD = chronic obstructive pulmonary disease; GOLD = Global Obstructive Lung Disease; mMRC = modified Medical Research Council scale; NYHA = New York Heart Association. *p*-values were tested by analysis of variance if the variable was continuous or by chi-square test if the variable was categorical (%).

*Data are expressed as mean ± SD or number (percentage).

^‡^ Significance level for comparisons vs. control group: p < 0.01;

^#^ *p* < 0.001

^§^ Significance level for comparisons vs. small airway dysfunction group: p < 0.01

Lung function characteristics of the three study groups are shown in Table 2. The respiratory resistances at 5 and 20 Hz were higher in patients with AL or SAD without AL than in the control group. Moreover, R5 also proved to be increased in patients with SAD without AL than in patients with AL. Therefore, the R5-R20 difference was increased in the SAD without AL group with respect to the other two, evidencing the existence of frequency dependence of respiratory resistance, characteristic of peripheral airway dysfunction. Moreover, the reactance, reactance area and resonant frequency were also higher in the patients with AL or SAD without AL than in control subjects, while the X5 was increased in patients with SAD without AL compared to the patients with AL. In turn, differences in spirometric parameters between control subjects and patients with SAD without AL were also detected. Regarding the former, the SAD without AL group had lower values of pre- and post-bronchodilator FVC and FEV_1_ as well as a decreased post-bronchodilator FEV_1_/FVC (z-score), despite staying in the normal range.

**Table 2:** Comparison of oscillometric and spirometric parameters between the study groups*.

	Control group	Small airway dysfunction group	Airflow limitation group	*p*-value
R5, kPa/l/s	0.38 (0.31–0.42)	0.67 (0.57–0.84) #	0.61 (0.42–0.72) #§	<0.001
R20, kPa/l/s	0.32 (0.28–0.36)	0.53 (0.45–0.65) #	0.56 (0.47–0.58) #	<0.001
R5-R20, kPa/l/s	0.05 (0.03–0.07)	0.12 (0.10–0.20) #	0.04 (0.02–0.06) §	<0.001
X5, kPa/l/s	0.33 (0.29–0.38)	0.56 (0.49–0.70) #	0.48 (0.38–0.60) #§	<0.005
AX, kPa/l	0.34 (0.22–0.54)	1.43 (0.86–2.44) #	1.06 (0.24–2.08) #	<0.001
Resonant frequency, 1/s	14.62 (13.11–16.40)	20.76 (17.79–24.87) #	19.66 (13.25–24.98) ‡	<0.001
Pre-bronchodilator FVC, l	4.000 ± 0.74	3.38 ± 0.82 #	4.16 ± 0.77 !!	<0.001
Pre-bronchodilator FVC, z-score	-0.55 ± 1.01	-1.09 ± 0.85 †	-0.33 ± 1.08 §	0.011
Pre-bronchodilator FEV_1_, l	2.98 ± 0.64	2.42 ± 0.63 #	2.39 ± 0.62 #	<0.001
Pre-bronchodilator FEV_1_, z-score	-0.78 ± 1.10	-1.54 ± 0.81 ‡	-2.00 ± 0.88 #	<0.001
Pre-bronchodilator FEV_1_/FVC	0.74 ± 0.06	0.73 ± 0.07	0.57 ± 0.09 #!!	<0.001
Pre-bronchodilator FEV_1_/FVC, z-score	-0.50 ± 0.81	-0.85 ± 0.97	-2.66 ± 0.76 #!!	<0.001
Post-bronchodilator FVC, l	4.07 ± 0.75	3.45 ± 0.82 #	4.27 ± 0.66 !!	<0.001
Post-bronchodilator FVC, z-score	-0.44 ± 1.00	-1.00 ± 0.81 ‡	-0.13 ± 1.00 §	0.003
Post-bronchodilator FEV_1_, l	3.15 ± 0.64	2.57 ± 0.68 #	2.56 ± 0.58 #	<0.001
Post-bronchodilator FEV_1_, z-score	-0.42 ± 1.11	-1.23 ± 0.88 #	-1.67 ± 0.91 #	<0.001
Post-bronchodilator FEV_1_/FVC	0.78 ± 0.07	0.76 ± 0.07	0.59 ± 0.08 #!!	<0.001
Post-bronchodilator FEV_1_/FVC, z-score	0.05 ± 0.95	-0.40 ± 1.06 †	-2.41 ± 0.70 #!!	<0.001

*Definition of abbreviations*: AX = area index of low frequent reactance; FEV_1_ = forced expiratory volume at 1 second; FVC = forced vital capacity; pred. = predicted; R20 = respiratory resistance at 20 Hz; R5 = respiratory resistance at 5 Hz; X5 = distal capacitive reactance at 5 Hz. p-values were tested by analysis of variance.

* Values are mean ± standard deviation or median (interquartile range), according their distribution.

^†^ Significance level for comparisons vs. control group: p < 0.05;

^‡^ p < 0.01;

^#^ p < 0.001.

^§^ Significance level for comparisons vs. small airway dysfunction group: p < 0.01;

^!!^ p < 0.001

As occurs with AL, the presence of SAD without AL also seems to have some degree of impact on quality of life, systemic inflammation and future health risk. Patients with SAD without AL had a quality of life that was poorer than control subjects and similar to that of patients with AL, both in the score of the COPD Assessment Test and in the physical component summary of the SF-12 questionnaire as well as in several domains of the same (Table 3). Similarly, subjects with AL or SAD without AL showed more systemic inflammation than the control group, reflected by a higher level of C-reactive protein. Finally, patients with AL or SAD without AL also had an increased cardiovascular risk and higher vascular age than control subjects. Regarding the use of healthcare resources, only a higher percentage of emergency room visits was detected in the AL group compared to the other two groups.

**Fig 2:**
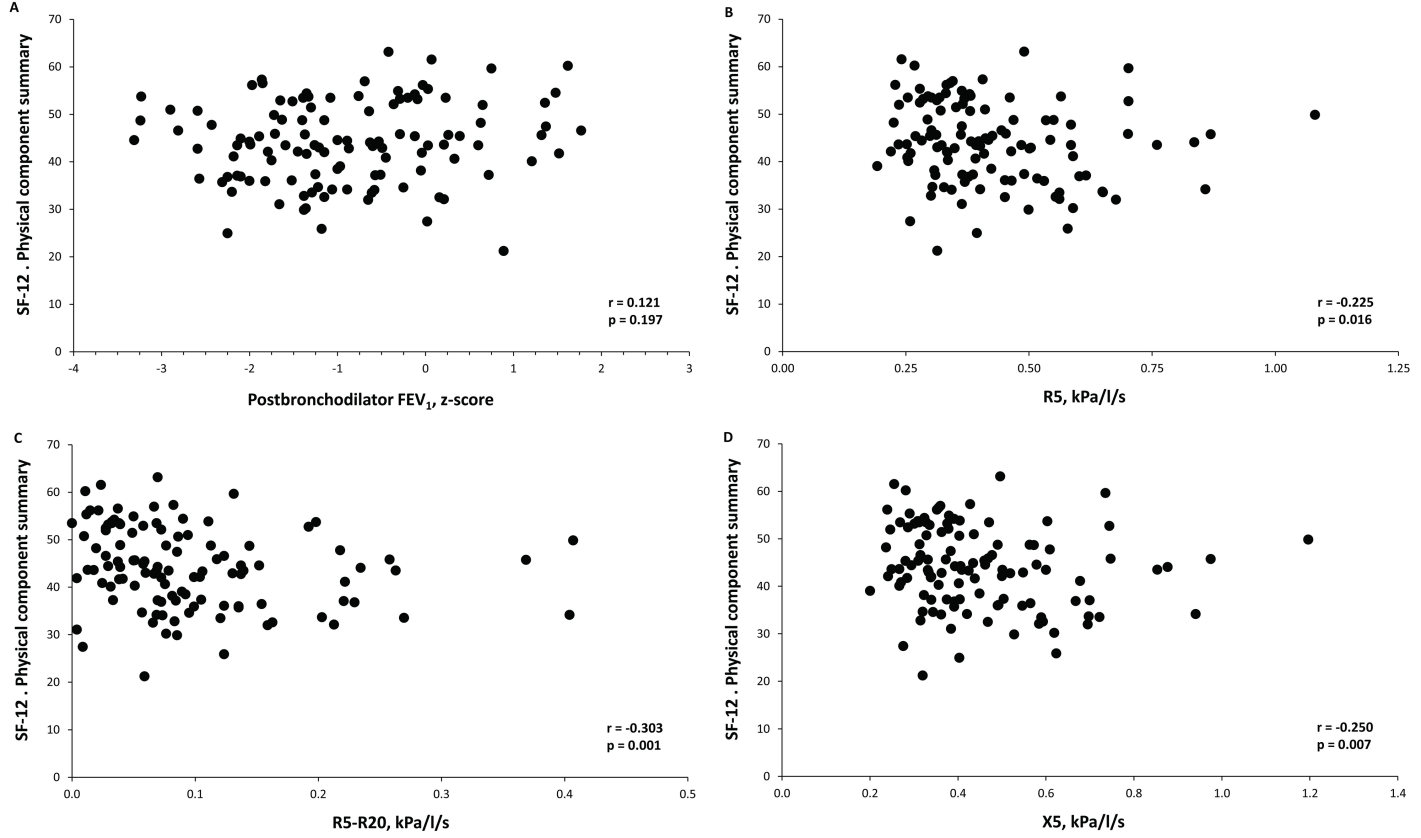
Relationship between the postbronchodilator forced expiratory volume at 1 second (FEV_1_) (A), the respiratory resistance at 5 Hz (R5) (B), the difference of the respiratory resistance at 5 Hz and 20 Hz (R5-R20) (C) and the reactance at 5 Hz (X5) (D) with the physical component summary of the SF-12 questionnaire in all study subjects.

**Fig 3:**
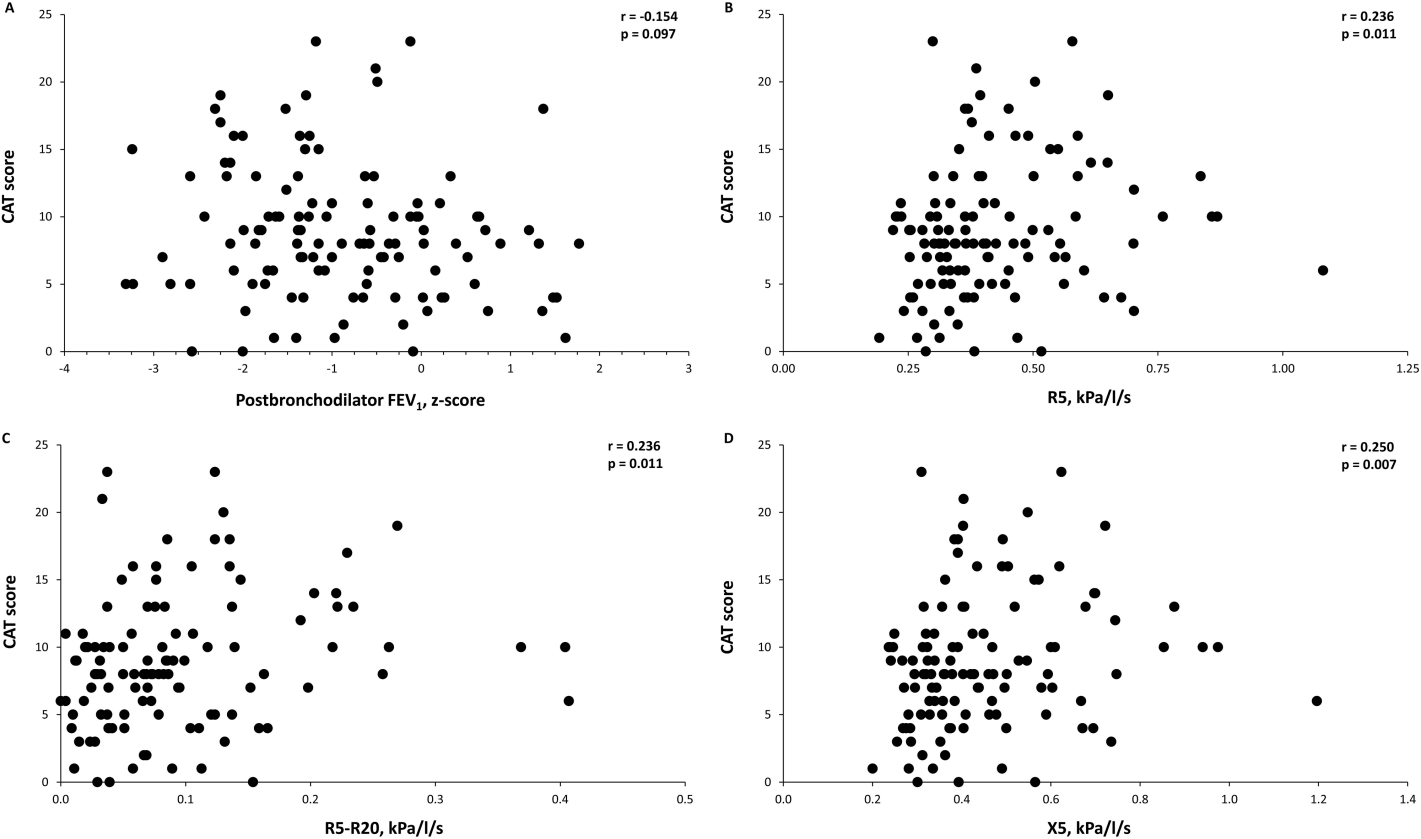
Relationship between the postbronchodilator forced expiratory volume at 1 second (FEV_1_) (A), the respiratory resistance at 5 Hz (R5) (B), the difference of the respiratory resistance at 5 Hz and 20 Hz (R5-R20) (C) and the reactance at 5 Hz (X5) (D) with the score of COPD Assessment Test in all study subjects.

In all study subjects, a relationship between oscillometric parameters of peripheral airway dysfunction and health-related quality of life was observed, whereas no relation was appreciated for spirometric values. R5, R5-R20 and X5 were inversely correlated with the physical component summary of the SF-12 questionnaire (Fig 2) and directly correlated with the COPD Assessment Test score (Fig 3). Moreover, when we analyzed separately patients without airflow limitation to exclude the potential inflammatory effects of airway obstruction, we found that the oscillometric parameters maintained a relationship with systemic inflammation. In fact, in this subgroup of patients, the C-reactive protein level was also directly related to R5, R5-R20 and X5 (S1 Fig).

**Table 3:** Comparison of health-related quality of life, systemic biomarkers, future health risk and health resources utilization between the study groups*.

	Control group	Small airway dysfunction group	Airflow limitation group	p-value
COPD Assessment Test	6.6 ± 4.3	9.3 ± 4.9 †	10.4 ± 5.2 ‡	0.006
SF-12 questionnaire				
Physical functioning	47.9 ± 9.0	42.8 ± 8.8 †	42.2 ± 8.4 ‡	0.018
Role (limitation) physical	52.6 (48.0–57.2)	57.2 (43.4–57.2)	48.0 (43.4–57.2)	0.551
Bodily pain	57.4 (47.3–57.4)	57.4 (57.4–57.4)	57.4 (47.3–57.4)	0.807
General health	45.2 ± 5.9	40.5 ± 8.0 †	38.4 ± 9.3 ‡	0.003
Vitality	54.0 ± 9.1	56.5 ± 8.9	51.5 ± 10.2	0.143
Social functioning	56.6 (36.4–56.6)	56.6 (46.5–56.6)	51.5 (46.5–56.6)	0.669
Role (limitation) emotional	44.9 (33.7–56.1)	56.1 (44.9–56.1)	44.9 (36.5–56.1)	0.295
Mental health	49.7 ± 9.2	53.1 ± 10.4	49.8 ± 9.5	0.246
Physical component summary	47.8 ± 8.7	42.9 ± 8.9 †	42.2 ± 7.2 †	0.017
Mental component summary	52.9 ± 9.3	48.6 ± 10.2	49.5 ± 10.3	0.336
BOD index	0.08 ± 0.27	0.13 ± 0.34	0.29 ± 0.46 ‡	0.035
ADO index	1.19 ± 0.98	1.59 ± 0.82	1.54 ± 0.93	0.122
Cholesterol, mg/dl	141 ± 34	155 ± 37	153 ± 30	0.111
LDL-cholesterol, mg/dl	75 ± 28	89 ± 39	84 ± 25	0.105
HDL-cholesterol, mg/dl	39 ± 10	45 ± 16	43 ± 10	0.073
Triglycerides, mg/dl	148 ± 130	142 ± 94	133 ± 70	0.847
Glycated hemoglobin, %	6.0 ± 1.2	5.9 ± 0.4	5.9 ± 0.5	0.903
hs-CRP, mg/L	1.52 (0.86–3.24)	2.64 (1.37–4.90) †	2.23 (0.84–5.91) †	0.024
pro-BNP, pg/ml	127 (69–285)	114 (48–302)	178 (88–429)	0.601
Fibrinogen, μmol/l	427 ± 110	440 ± 122	501 ± 141 †	0.037
Cardiovascular disease risk, %	17.3 ± 9.5	21.3 ± 8.2 †	22.8 ± 8.4 †	0.044
Heart/vascular age, years	66.2 ± 12.7	71.0 ± 8.7 †	72.3 ± 9.9 †	0.048
Health resources utilization in the last year				
Hospitalization, n (%)	27 (43%)	13 (43%)	14 (58%)	0.406
Emergency Room visit, n (%)	18 (29%)	8 (27%)	14 (58%)	0.020

*Definition of abbreviations*: ADO = age, dyspnea and airway obstruction; BNP = brain-type natriuretic peptide; BOD = body mass index, airway obstruction and dyspnea; COPD = chronic obstructive pulmonary disease; CVD = cardiovascular disease; HDL = high-density lipoprotein; hs-CRP = high-sensitivity C-reactive protein; LDL = low-density lipoprotein; SF-12 = Short Form 12. p-values were tested by analysis of variance if the variable is continuous or by chi-square test if the variable is categorical.

*Values are mean ± standard deviation, median (interquartile range) or number (percentage) according their distribution.

^†^ Significance level for comparisons vs. control group: p < 0.05;

^‡^ p < 0.01.

Finally, using a multivariate stepwise logistic regression model, we identified the following variables as being independently associated with the presence of SAD without AL versus normal lung function: elevated pack-years, as well as higher levels of HDL cholesterol and hs-CRP (Table 4).

**Table 4:** Independent risk factors for small airway dysfunction versus normal lung function*.

	Adjusted odds ratio	95% confidence interval	p-value
Pack-years	1.025	1.002 to 1.049	0.030
HDL-cholesterol, mg/dl	1.076	1.014 to 1.142	0.015
log (hs-CRP)	5.963	1.1732 to 30.320	0.031

*Definition of abbreviations*: HDL = high-density lipoprotein; hs-CRP = high-sensitivity C-reactive protein; log = logarithm.

*Multivariate stepwise logistic regression analysis adjusted for sex, age, body mass index, Canadian Cardiovascular Society grade of angina and New York Heart Association functional class.

## Discussion

Our study shows that, in smoking patients with IHD, small airway dysfunction is even more prevalent than airflow limitation, at rates of 26.3 and 20.3%, respectively. The presence of SAD, which is independently related with smoking intensity and plasma levels of HDL-cholesterol and CRP, is associated with reduced health status and increased future cardiac risk.

The prevalence of airflow limitation detected in our study was lower than that recently observed in the ALICE study (30.5%), although it is within the range observed across participating countries of 18.3 to 41.3% [8]. In addition to the geographical variation, we believe that this difference is mainly explained by the fact that this present study has used a stricter criterion to define airflow limitation by incorporating the lower limit of normal to the established criterion of post-bronchodilator FEV_1_/FVC <0.7, in accordance with current international guidelines for interpreting lung function [24].

Another important methodological aspect has to do with the criteria used to identify SAD. This diagnosis was established when the R5 and the R5-R20 difference surpassed the upper limit of normal, to detect increased resistance of the peripheral airways and the frequency dependence of specific airway resistance, which are characteristic of SAD [12]. To avoid false positives, reactance at 5 Hz was not used as it has been reported that up to 11% of patients with IHD can have a restrictive disorder [8]. Since the reactance or “rebound resistance” represents capacitance or the ability to store energy, it depends on peripheral airway function, but also on the dimensions of the ventilated airways and elasticity of the lungs and thorax [12]. In patients with restrictive lung disorders, there have been reports of increased reactance, especially at low frequencies, with small, not significant changes in respiratory resistance [25]. Moreover, in patients with left ventricular failure, an increase of the respiratory reactance that correlated with orthopnea severity and improved after administering nitrates has been published [26].

Our study demonstrates that SAD is frequent in smoking patients with IHD, representing 26.3% of the total and 33% of those with normal spirometry. In order to properly evaluate these percentages, one must consider the report that 5–10% of smokers with normal spirometry had “abnormal” IOS [27]. Similarly, other authors have reported that the prevalence of SAD in healthy current or former smokers with normal spirometry ranges from 6–13% [28]. In situations with massive exposure to other irritating substances, such as in World Trade Center workers, the prevalence of an elevated R5-R20 difference ranges from 10.9 to 14.4% [22].

Among the factors associated with the presence of SAD, we have identified smoking intensity and elevated levels of HDL-cholesterol and CRP (Table 4). It is well known that oxidative stress and direct damage caused by the inhalation of tobacco smoke leads to the progressive infiltration of the small airways by polymorphonuclear neutrophils, macrophages, CD4 cells and lymphocyte subtypes, as well as thickening of the airway walls and accumulation of inflammatory exudates in the lumen [9,29].

The presence of elevated levels of HDL-cholesterol was also identified as a factor independently associated with SAD. Interestingly, higher HDL levels have been associated with lower FEV_1_/FVC ratio and a higher percentage of emphysema, suggesting a role for the apolipoprotein M/HDL pathway in the pathogenesis of distal airway damage [30]. In fact, it has been demonstrated that HDL inhibits tumour necrosis factor-stimulated sphingosine kinase activity in human endothelial cells, thereby increasing ceramide and decreasing sphingosine-1-phosphate (S1P) cellular levels [31]. Ceramide, a second messenger molecule, modulates endothelial cell apoptosis, while S1P has an essential role in maintaining endothelial barrier integrity in the lung, and both are implicated in small airway damage and emphysema pathogenesis [30].

Finally, an independent association between high levels of hsCRP and the presence of SAD was also identified. There is much evidence to suggest that increased innate immune responsiveness, characterized by elevated systemic inflammatory biomarkers like C-reactive protein, particularly in those who have smoked, may contribute directly to the development of obstructive diseases. Large prospective studies have described that chronically elevated CRP is associated with a progressive loss of lung function and greater risk of airflow limitation [32,33]. Moreover, it has been proposed that these circulating biomarkers might maintain the activation of neutrophils and macrophages in both the circulation and lungs of susceptible smokers [34]. At any rate, our data cannot exclude the participation of other factors in the development of SAD, such as interstitial edema secondary to ventricular failure [35], the use of beta-blockers with a recognized bronchoconstrictor effect on the small airways [36], certain infections like viral bronchiolitis [37], or even air pollution [38].

In addition to its prevalence, the presence of SAD without AL in smokers with IHD is clinically relevant as it is associated with poorer lung function, poorer health-related quality of life and higher future cardiac risk, in addition to the suggestion of its being a stage prior to the development of COPD or cardiac asthma [9,29]. It has been reported that airflow limitation is independently predicted by small airways disease and that SAD contributes to COPD severity in an independent manner [39]. In fact, several evidences indicate that inflammation with fibrosis, wall thickening, and mucus in the small airways cause narrowing and reduction in numbers of terminal bronchioles, that contribute to the rapid decline in FEV_1_ reflecting the progressive airway obstruction in COPD [9,40]. Moreover, the observation that terminal bronchiolar loss precedes the onset of emphysematous destruction suggests this destruction begins in the very early stages of COPD [29]. Therefore, even when spirometry is normal, dysfunction of small airways is a sensitive marker of disease [39]. These results concur with the recent demonstration that symptomatic current or former smokers without airflow limitation have more exacerbations, activity limitation, and evidence of airway dysfunction than control subjects who had never smoked [41].

Taken together, our results demonstrate the need to complement spirometry with other procedures to evaluate airway function in smokers with IHD [42]. Although oscillometric parameters reach a significant relationship with quality of life and systemic inflammation, which is not the case with FEV_1_, these relationships are weak, probably due to the limited sample size and the intrinsic variability of these measurements. For this reason, the choice of an isolated oscillometric parameter to the assessment of individual patients does not seem advisable, but rather an integrated interpretation of the IOS to identify the existence of SAD.

Our study has several limitations. Due to its single-center nature and limited simple size, the results obtained should be extrapolated with caution, and later verification is necessary. At the same time, as it is a cross-sectional study and does not have a longitudinal design, causal relationships cannot be established. In addition to the variables analyzed, it would be prudential to assess the impact of the presence of SAD in smoking patients with IHD on more clinically relevant outcomes, such as mortality or new episodes of myocardial infarction.

## Conclusion

Our study demonstrates that SAD without AL is frequent in smoking patients with IHD and associated with poorer clinical condition and greater future cardiac risk. Because SAD is a precursor to the development of COPD, and because of the possibility to offer these patients respiratory treatments, the evaluation of small airway function in smoker IHD patients could be considered by using a quick, simple and non-invasive procedure such as impulse oscillometry.

## Supporting information

S1 Fig Lung function and inflammation in patients without airflow limitation. Relationship between the postbronchodilator forced expiratory volume at 1 second (FEV_1_) (A), the respiratory resistance at 5 Hz (R5) (B), the difference of the respiratory resistance at 5 Hz and 20 Hz (R5-R20) (C) and the reactance at 5 Hz (X5) (D) with the high-sensitivity C-reactive protein (hsCRP) in patients without airflow limitation.
(PDF)

## Abbreviations

AL: airflow limitation
AX: reactance area
CAT: COPD Assessment Test
CCS: Canadian Cardiovascular Society
COPD: chronic obstructive pulmonary disease
CRP: C-reactive protein
HbA1c: glycated haemoglobin
HDL: high-density lipoprotein
IHD: ischemic heart disease
IOS: impulse oscillometry system
MRC: Medical Research Council
NYHA: New York Heart Association
pro-BNP: pro-brain natriuretic peptide
R20: resistance at 20 Hz
R5: resistance at 5 Hz
SAD: small airway dysfunction
X5: reactance at 5 Hz
