# Supplementary material for: Small airway dysfunction in smokers with stable ischemic heart disease
Source: PLoS One. 2017 Aug 28;12(8):e0182858. doi: 10.1371/journal.pone.0182858 (PMC5573211; doi:10.1371/journal.pone.0182858)
Supplement: S1 Fig — Relationship between the postbronchodilator forced expiratory volume at 1 second (FEV1) (A), the respiratory resistance at 5 Hz (R5) (B), the difference of the respiratory resistance at 5 Hz and 20 Hz (R5-R20) (C) and the reactance at 5 Hz (X5) (D) with the high-sensitivity C-reactive protein (hsCRP) in patients without airflow limitation. (PDF) [file pone.0182858.s001.pdf]

Supporting information

Title: Small airway dysfunction in smokers with stable ischemic heart disease

Authors: Claudia Llontop[1], Cristina Garcia-Quero[2], Almudena Castro[3], Regina Dalmau[3], Raquel Casitas[2,4], Raúl Galera[2,4], Alberto Iglesias[2], Elisabet Martinez-Ceron[2,4], Joan B Soriano[5,6], Francisco García-Río[2,4,6]

S1 Fig. Lung function and inflammation in patients without airflow limitation.

Relationship between the postbronchodilator forced expiratory volume at 1 second (FEV1) (A), the respiratory resistance at 5 Hz (R5) (B), the difference of the respiratory resistance at 5 Hz and 20 Hz (R5-R20) (C) and the reactance at 5 Hz (X5) (D) with the high-sensitivity C-reactive protein (hsCRP) in patients without airflow limitation

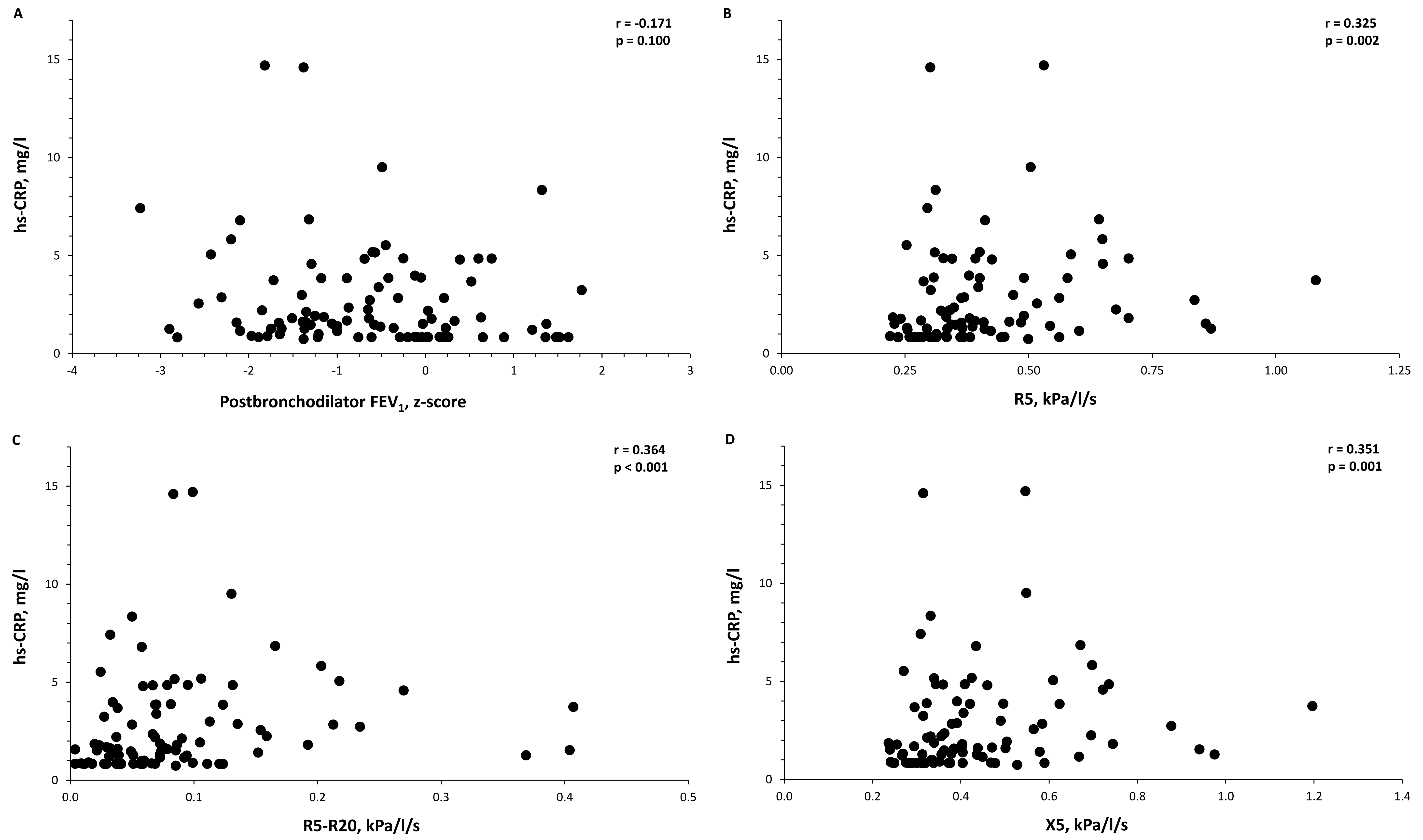

A
r = -0.171
p = 0.100
hs-CRP, mg/l
Postbronchodilator FEV_1, z-score
B
r = 0.325
p = 0.002
hs-CRP, mg/l
R5, kPa/l/s
C
r = 0.364
p < 0.001
hs-CRP, mg/l
R5-R20, kPa/l/s
D
r = 0.351
p = 0.001
hs-CRP, mg/l
X5, kPa/l/s
